# Large-scale SNP discovery and construction of a high-density genetic map of *Colossoma macropomum* through genotyping-by-sequencing

**DOI:** 10.1038/srep46112

**Published:** 2017-04-07

**Authors:** José de Ribamar da Silva Nunes, Shikai Liu, Fábio Pértille, Caio Augusto Perazza, Priscilla Marqui Schmidt Villela, Vera Maria Fonseca de Almeida-Val, Alexandre Wagner Silva Hilsdorf, Zhanjiang Liu, Luiz Lehmann Coutinho

**Affiliations:** 1 Animal Science department, University of São Paulo (USP)/Luiz de Queiroz College of Agriculture (ESALQ), Piracicaba, São Paulo, Brazil; 2 The Fish Molecular Genetics and Biotechnology Laboratory, Aquatic Genomics Unit, School of Fisheries, Aquaculture and Aquatic Sciences and Program of Cell and Molecular Biosciences, Auburn University, Auburn, AL, 36849, United States of America; 3 Nature and Culture Institute, Federal University of Amazon (UFAM), Benjamin Constant, Amazonas, Brazil; 4 Unit of Biotechnology, University of Mogi das Cruzes, P.O. Box 411, 08701-970, Mogi das Cruzes, SP, Brazil; 5 Brazilian National Institute for Research of the Amazon, Laboratory of Ecophysiology and Molecular Evolution, Manaus, Amazonas, Brazil; 6 University Nilton Lins, Aquaculture Graduate Program, Manaus, Amazonas, Brazil

## Abstract

*Colossoma macropomum*, or tambaqui, is the largest native Characiform species found in the Amazon and Orinoco river basins, yet few resources for genetic studies and the genetic improvement of tambaqui exist. In this study, we identified a large number of single-nucleotide polymorphisms (SNPs) for tambaqui and constructed a high-resolution genetic linkage map from a full-sib family of 124 individuals and their parents using the genotyping by sequencing method. In all, 68,584 SNPs were initially identified using minimum minor allele frequency (MAF) of 5%. Filtering parameters were used to select high-quality markers for linkage analysis. We selected 7,734 SNPs for linkage mapping, resulting in 27 linkage groups with a minimum logarithm of odds (LOD) of 8 and maximum recombination fraction of 0.35. The final genetic map contains 7,192 successfully mapped markers that span a total of 2,811 cM, with an average marker interval of 0.39 cM. Comparative genomic analysis between tambaqui and zebrafish revealed variable levels of genomic conservation across the 27 linkage groups which allowed for functional SNP annotations. The large-scale SNP discovery obtained here, allowed us to build a high-density linkage map in tambaqui, which will be useful to enhance genetic studies that can be applied in breeding programs.

Tambaqui (*Colossoma macropomum*) is the most important native aquaculture species in Brazil. In 2014, production reached 139,209 tons^1^. This species has been broadly farmed in different Amazon boundary countries and has been introduced to other Latin American countries^2^, as it is well-suited to aquaculture farming, accepts artificial feed, grows rapidly, and is accepted by consumer markets^3^. However, genetic studies of tambaqui are limited to genetic diversity surveys^4^,^5^,^6^, transcriptome analysis under specific conditions^7^, some SNPs markers^8^, and mitochondrial genome sequencing^9^.

Genetic linkage maps are essential resources for genomic and genetic research. They provide frameworks for understanding genome structure and function. Linkage maps are essential for quantitative trait locus (QTL) identification and mapping. QTL mapping is an important way to identify genes that are related to trait variations within and between populations or species, allowing for implementation of genetic and breeding programs^10^. Genetic maps can be used to facilitate genome assembly corrections, anchoring scaffolds onto linkage groups to build chromosomal assembly^11^. With interspecific crosses, high-density genetic mapping can provide a genome-scan of segregation distortion within the genome^12^ and can investigate genomic incompatibilities between species at the genome level^13^. Genetic studies such as genetic linkage mapping^14^, QTL mapping^15^, populational genetic analysis^16^, and genome-wide association studies^17^ require a large number of reliable molecular markers across the genome.

Single nucleotide polymorphisms (SNPs) are the marker of choice for genetic studies in various organisms. SNPs are the most abundant molecular markers in any vertebrate genome, with a SNP present in 100–500 bp on average^18^. SNPs are mostly bi-allelic, making them amenable for high-throughput genotyping using SNP arrays^19^,^20^. Over the last decade, with the development of next-generation sequencing technologies^21^,^22^,^23^, genome-scale SNP markers can now be efficiently and cost-effectively identified in any organism for which prior genomic information does not yet exist^24^. Genotype-by-sequencing (GBS) is one next-generation sequencing technique, and is based on the reduction of genome complexity using restriction enzymes^23^. GBS is characterized as a simple, quick, specific, reproducible technique^23^, and has been extensively used to identify a large number of SNPs in various model and non-model species^25^,^26^,^27^,^28^.

In the present study, we applied GBS to identify large-scale SNPs and construct a high-density genetic linkage map for tambaqui, using the Illumina HiSeq 2500 platform. In addition, we conducted syntenic relationship and functional annotation analyses by aligning tambaqui against the zebrafish (*Danio rerio*) genome. This study provides large-scale SNP markers and high-density linkage maps in tambaqui, which can be a useful resource for facilitating the tambaqui physical map construction, genome assembly, and QTL mapping to enhance genetic studies and breeding programs.

## Results

### Enzyme selection

Based on *in silico* and *in vitro* genomic fragmentation, we tested whether the enzymes *PstI* and *SbfI* would generate the expected number of reads required to obtain ~7X sequencing coverage^29^. Each enzyme generated a different distribution of fragment lengths across the entire genome (Supplementary Figure S1). The enzyme *PstI* yielded a larger number of fragments that ranged between 200 bp and 500 bp (see Supplementary Figure S1), providing suitable sizes for GBS to be clustered on cBOT (Illumina) in bridge amplification.

### Sequencing

As summarized in Table 1, the 126 samples generated over 352 million single-end reads that had a length of 100 bp. After read trimming, ~285 million quality reads (81%) were obtained, with over 27 Giga bases, equivalent to >8X genome coverage. An average of 3.2 million quality reads were obtained from the parents and over 2.2 million quality reads were obtained from each progeny (Table 1). The vast majority of samples had genome sequencing depth >8X, and only 15 progenies had fewer than one million reads (~4.5X) (Supplementary Spreadsheet S1).

### SNP discovery

A total of 81,222 pairwise alignments were obtained with the UNEAK pipeline. After filtering with default parameters, 68,584 putative SNPs that had a minor allele frequency (MAF) that was greater than 0.05 (Table 2) were identified. SNPs were classified into transitions (Ti) and transversions (Tv) based on nucleotide substitution. The number of A/G transitions was about two times greater than C/T transitions; the numbers of A/C and A/T transversions were relatively higher than the C/G and G/T transversions. Transitions are the most common type of nucleotide substitutions, and in this sample 64% of the base changes were transitions and 36% were transversions, with an observed transition to transversion ratio (Ti/Tv) of 1.8:1.

### SNP filtering

A set of 10,288 high quality SNP markers that had a sample call rate >80% and that were heterozygous for at least one parent were retained for further analysis. After removing markers with significant segregation distortion, a total of 7,734 SNPs were retained for genetic map construction. Of these SNPs, 3,641 were heterozygous only in female, 2,565 were heterozygous only in male, and the remaining 1,528 were heterozygous in both parents. A total of 118 samples with a SNP call rate >80% were retained for genetic map construction.

### Construction of the high-density linkage map

**Figure 1:**
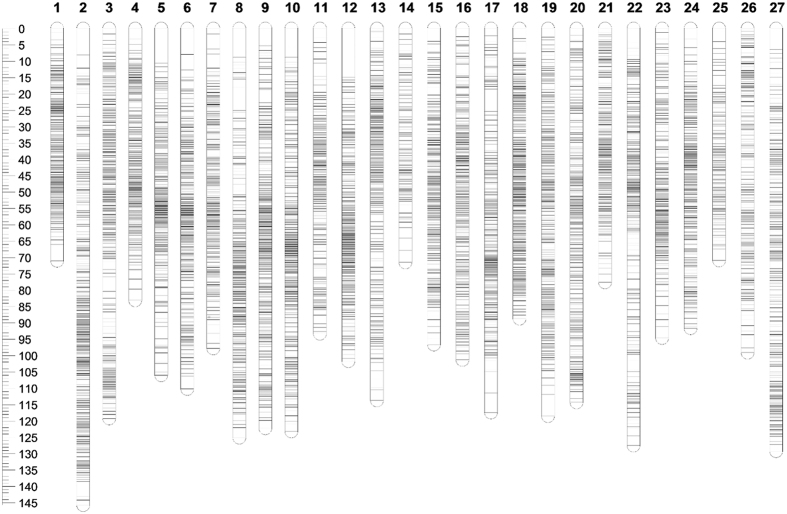
Graphical presentation of the tambaqui genetic linkage map. The 27 linkage groups are represented by vertical bars while the horizontal lines represent markers. Genetic distances between adjacent markers are shown on the side ruler in centiMorgan (cM).

A total of 7,192 SNPs were mapped to 27 linkage groups, consistent with tambaqui’s haploid chromosome number (n = 27)^30^ (Supplementary Spreadsheet S2). The genetic map spanned a total of 2,810.9 cM, with an average marker-interval of 0.39 cM (Fig. 1). On average, each linkage group contained 266 markers that spanned an average length of 104 cM, with an average marker interval of 0.41 cM. The number of mapped markers per linkage group varied from 86 markers on LG14 to 362 markers on LG2 and LG9. The smallest linkage group was LG25, which contained 120 markers spanning a length of 70.94 cM. The largest linkage group was LG2, which had 362 markers and a length of 145.66 cM. The maximum gap size in each linkage group ranged from 2.08 cM on LG20 to 14.79 cM on LG12, with an average of 5.27 cM (Table 3).

**Figure 2:**
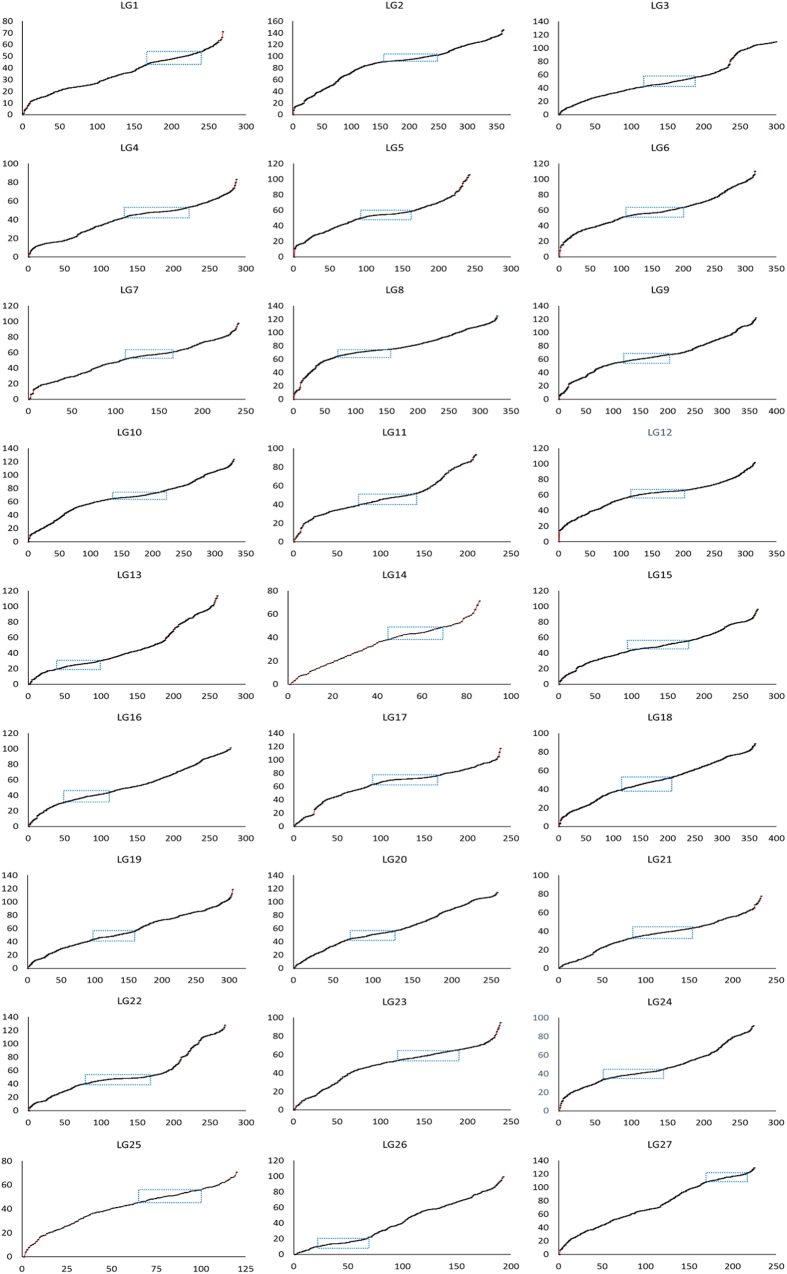
Patterns of marker distribution along the tambaqui linkage groups. The dotted box indicates regions with higher marker density. The red lines indicate gaps without markers.

The distribution of markers across each linkage group was assessed using the sliding window approach. The number of markers within a window was counted using a sliding window of 10 cM with a step size of 1 cM. The density value for each window was calculated by dividing the total of markers within a window by the window length. As shown in Fig. 2, the SNP markers are evenly distributed across the 27 linkage groups. The linkage groups LG2, LG4, LG6, LG8, LG9, LG12, and LG22 had windows with high density of markers (>8 markers per cM), most of which were clustered at the same genetic positions. LG14 had more windows with low density of markers. LG12 had the window with the highest density (10.4 markers per cM) and also the window with the lowest density (0 markers per cM). In general, the regions with high marker density were located near the centromeres (Fig. 2).

### Comparative genomic analysis

**Figure 3:**
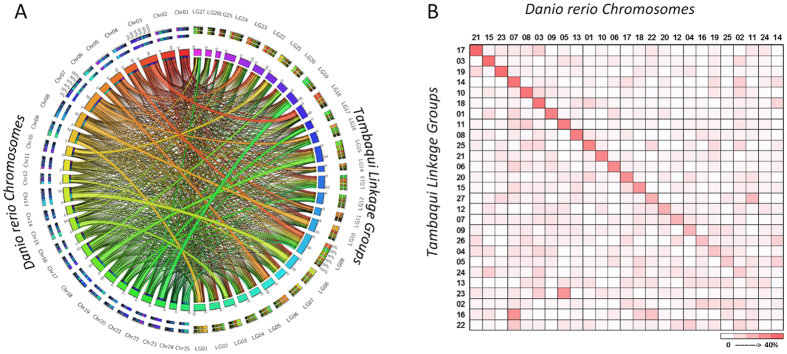
Comparison between the tambaqui linkage map and the zebrafish genome. Syntenic links between tambaqui linkage map and zebrafish genome were made with the Circos software (**A**). Plot of the percentage of SNPs that are shared between tambaqui linkage groups and zebrafish chromosomes (**B**). In synteny comparison, the connecting links are color coded according to the zebrafish chromosomes arc to their tambaqui linkage-group corresponding.

Out of the 7,192 SNP markers mapped to the linkage map, 1,237 markers that contained sequences were successfully aligned against the zebrafish genome (Fig. 3). The synteny analysis between tambaqui and zebrafish showed variable levels of genomic conservation across the 27 linkage groups. Most tambaqui linkage groups showed a relationship with homologous zebrafish chromosomes. Through comparative analysis, a high level of genomic conservation was found between tambaqui and zebrafish for most linkage groups. For instance, more than 30% of the SNPs mapped in LGs 17, 3, 19, 14, 10, 18, 1, 11, 8, 25, 21, 6, 20, and 15 of tambaqui were aligned on zebrafish chromosomes 21, 15, 23, 7, 8, 3, 9, 5, 13, 1, 10, 6, 17, and 18, respectively, suggesting orthologous chromosomal relationships. Zebrafish chromosome 7 corresponded to tambaqui LGs 14 and 16, while zebrafish chromosome 5 corresponded to LGs 11 and 23 in tambaqui. LG 27 in tambaqui had a similar level of consensus between homologous chromosomes 11 and 22 in zebrafish. However, LGs 2, 4, 5, 13, 22, 24, and 26 in tambaqui appear scattered among several chromosomes, suggesting large-scale chromosomal rearrangements between species.

### Functional annotation

The SNPs for each linkage group were annotated against the genes from the zebrafish (ENSEMBL release 84). This annotation allowed us to evaluate the potential use of our genetic map with respect to possible important traits in breeding programs. Approximately 60% (742) of the SNPs that were evaluated were annotated to be intronic (36%), downstream (13%), or upstream (11%) of gene regions. These SNPs could thus have a direct effect on or be associated with potential performance traits of interest (Table 4). The other 40% were evaluated as synonymous variant (11%), intergenic variant (10%), missense variant (7%), non-coding transcript variant (4%), splice region variant (3%), 3 prime UTR variant (1%) and other variants (4%). Only 2% of the SNPs resulted in a stop lost codon, and 1% results in a stop gain codon, both of which can lead to protein truncation (Supplementary Spreadsheet S3).

### Allele frequency and coverage evaluation

**Figure 4:**
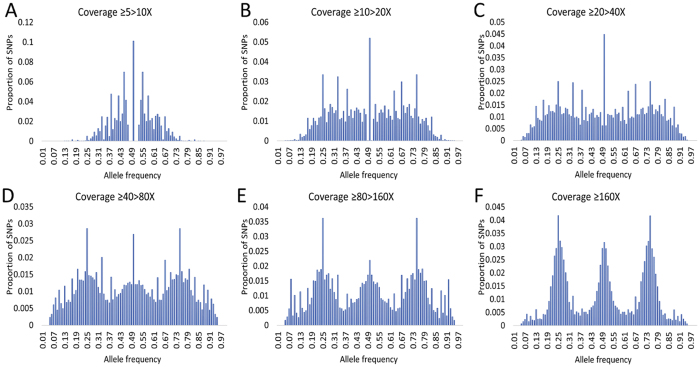
Effect of coverage depth on allele frequency of tambaqui (*Colossoma macropomum*) genotyped with GBS.

To evaluate the GBS approach’s capability in determining the accuracy of allelic frequencies, we compared the allelic frequency obtained in our genotyping with the frequency expected for a diploid cross. We also analyzed allele frequency calls into population using different SNP coverage thresholds (Fig. 4). The SNPs were placed into six groups, depending on coverage (5–10X, 10–20X, 20–40X, 40–80X, 80–160X, ≥160X). For SNPs with coverage ≥160X, we observed three peaks in allele frequency distribution. The first and third peak represent the crosses of AA x Aa, with allele frequencies of 0.25 and 0.75, respectively, with a 1:1 segregation of the AA and Aa genotypes. The second peak represents the cross Aa x Aa, with allele frequencies of 0.5 and a 1:2:1 segregation of the AA, Aa, and aa genotypes. Lower coverage resulted in allelic frequency deviations from expected frequencies.

## Discussion

Developing molecular marker panels and genetic linkage maps are essential for genetic improvement programs in aquaculture. Despite the economic and ecological importance of tambaqui, limited genomic resources are available. In this study, we report the first genome-scale SNP discovery and high-density genetic map construction in tambaqui. Sequencing the family under study allowed for the identification and genotyping of a large number of markers in an efficient and cost-effective way. The linkage mapping analysis resulted in the same number of linkage groups as corresponds to tambaqui’s haploid chromosome number (n = 27)^30^. In addition, this process created a high-density linkage map.

SNP discovery and genetic map construction using the GBS approach have been conducted in a number of aquaculture species including Asian seabass (*Lates calcarifer*)^31^, common pandora (*Pagellus erythrinus*)^32^, sablefish (*Anoplopoma fimbria*)^33^, scallop (*Chlamys farreri*)^34^, oysters (*Crassostrea gigas × Crassostrea angulata*)^34^, and small abalone (*Haliotis diversicolor*)^13^,^14^,^24^,^31^,^32^,^34^,^35^. This tambaqui genetic map will be valuable for genomic research and genetic enhancement applications, such as identifying interspecific hybrids^30^ and implementing genome-wide association studies^36^.

Successfully applying the GBS approach depends on choosing an effective restriction enzyme. This choice determines the number of genomic fragments produced by the complexity reduction for the species under study, which in turn are used to identify genome-wide sequence polymorphisms^37^. The *in silico* fragmentation showed that *PstI* and *SbfI* produce a large difference in the number of restriction sites. The *PstI* enzyme was more suitable for the fragmentation of the genome tested. This enzyme has also been successfully used in prawn kuruma (*Marsupenaeus japonicas)* for high-resolution genetic linkage map construction and QTL mapping^35^.

The per-base quality of sequencing reads has a great impact on the accuracy of marker detection and genotype calling^38^. In our analysis, we trimmed off 19% of all reads with low-quality scores. This decision resulted in 28% larger numbers of identified SNPs than that from non-trimed reads (unpublished data). This is because the UNEAK Pipeline performs SNP discovery based on a network of SNPs formed by reads that have only a base incompatibility. Low-quality reads create more complicated networks during SNP discovery; it also reduces read counts below a specified error tolerance rate of 0.03. The UNEAK Pipeline considers this networks as a result of sequencing errors and exclude them from genotyping^39^, reducing SNP coverage. In our analysis, SNP coverage had a direct effect on allelic frequency (Fig. 4). The results showed that higher SNP coverage results in an allele frequency distribution that is consistent with an expected diploid crossing. For instance, the coverage of ≥160X provided smaller levels of missing data and a higher percentage of called genotypes (Fig. 4).

GBS was an efficient method for discovering tens of thousands of SNPs in a genome that had no reference sequence. However, genetic maps require high-quality genotypes of markers from a certain number of mapping samples. In this study, we used high criteria to SNP call rate and sample call rate. Although the filtering steps eliminated 85% of the 68,584 SNPs, the remaining markers and samples had high-quality scores that ensured the construction of genetic map with a high level of accuracy. Similar numbers of makers were also reported in other studies using GBS strategies^31^,^34^,^40^,^41^, which generally show high levels of missing genotypes due to the relatively low sequencing depth.

The GBS approach generated marker-containing sequences, allowing for analysis with other closely-related model species based on sequence homology (Fig. 3). About 17% of the tambaqui SNPs were successfully aligned with the zebrafish genome. This percentage is higher than that reported in Mexican tetra (*Astyanax mexicanus*) (14.2%)^11^. However, this higher alignment could be a consequence of the genetic distance between the species used or a consequence of manually maximizing the Bowtie alignment parameters, since that our alignment, using the default parameter, falls from 17% to 9%. A variable degree of synteny is observed between tambaqui linkage groups and zebrafish chromosomes, with some linkages groups showing homology with several chromosomes of zebrafish. The majority of the linkage groups in tambaqui have one-to-one orthologous relationships with chromosomes zebrafish. Notably, zebrafish chromosomes 5 and 7 have two-to-one syntenic relationships with tambaqui linkage groups. The use of zebrafish to perform synteny and collinearity analyses may provide a framework for mapping candidate genes responsible for the traits related to phenotypic divergence in tambaqui. Zebrafish genome also enables the transfer of genomic information between zebrafish and others species, such as Mexican tetra^11^, gudgeons^42^, common carp^43^, rainbow trout^44^, and channel catfish^45^.

The syntenic relationships and functional annotation of zebrafish allowed for annotation of the SNPs from the tambaqui linkage map. In turn, this allowed for the identification of 1,237 variants, from which 36% were annotated in genomic regions (Supplementary Spreadsheet S3). Candidate genes for important traits in tambaqui were identified, such as insulin-like growth factor binding protein (*IGFBP*), a hypoxia-inducible gene that acts in regulating embryonic growth and development under hypoxic stress^46^. We also found variants in genes that are of medical interest, such as insulin-like growth factor 1a receptor (*IGF1RA*), a gene that has been studied in animals with relatively slow progression for insulin resistance to better understand possible genes or metabolic situations that may accelerate diabetes progression, thereby offering possible new therapeutic targets^47^. In addition, variants of upstream, downstream, and intronic regions of the immunoglobulin heavy chain variable (*IGHV*) gene were annotated. Interesting, this gene has been reported to be the most important indicator of chronic lymphocytic leukemia prognosis^48^. Although the tambaqui genome sequences are still not available, comparative genome analysis enabled the identification of large number of markers in genic regions, which in turn could be used in tambaqui breeding programs.

## Conclusion

This study demonstrated that GBS is an effective approach for SNP discovery and the development of high-density genetic linkage maps in tambaqui. This study was also the first to identify genome-scale SNP markers and to construct high-density genetic maps for tambaqui. Comparative genomic analysis with zebrafish revealed variable levels of homologous relationships with zebrafish chromosomes for all tambaqui linkage groups. In addition, large numbers of markers in genic regions were annotated, and genes with potential functions for performance traits were identified. The SNPs and genetic map reported in this work should be valuable tools for genetic studies and aquaculture improvements in tambaqui.

## Materials and Methods

### Ethical statement

All experimental protocols employed in the present study that relate to animal experimentation were performed in accordance with Brazilian Directive for the Care and Use of Animals in Teaching or Scientific Research Activities, resolution number 30/2016 approved by the National Animal Experimentation Control Council to ensure compliance with international guidelines for animal welfare.

The samples were acquired from a commercial hatchery and were not subjected to any experimental manipulation or euthanasia. No specific permits were required for the work described here.

### Fish materials and DNA extraction

The *Colossoma macropomum* subjects used in this study were collected at a fisheries farm located in the Rondônia state, in the northwestern region of Brazil. A full-sib F1 family of 124 offspring was created by crossing a wild female with a wild male. When the subject’s mean body weight reached ~6 g (mean body length of ~5.5 cM), tail fin clips were collected from each progeny and the two parents, and preserved in 90% ethanol. DNA extraction followed these steps: proteinase K digestion (Promega), DNA precipitation in absolute ethanol, washing in 70% ethanol, and resuspension in ultrapure water. DNA concentration was quantified using a Qubit 2.0 fluorimeter (Invitrogen, Carlsbad, CA, USA) and Nanodrop^®^2000c spectrophotometer. DNA integrity was checked in 1% agarose gel. All DNA samples were stored at −20 °C prior to sequencing library preparation.

### Construction of GBS sequencing libraries

To select an enzyme that uniformly distributed cutting sites across the tambaqui genome, we performed an *in-silico* DNA cleavage on the zebrafish genome with *PstI* and *SbfI* in R using the subsequent Bioconductor^49^ packages: Biostrings, BSgenome.Drerio.UCSC.danRer7, plyr, ggplot2, reshape2 and scales (https://github.com/). Additionally, we performed *in vitro* genomic cleavage of tambaqui DNA samples with the *PstI* and *SbfI* enzymes, according to the manufacturer’s protocol (New England BioLabs^®^).

GBS library construction and sequencing were conducted at the Animal Biotechnology Laboratory at the University of São Paulo (Piracicaba, Brazil), using the protocol described by Elshire *et al*.^23^ with modifications. In brief, 100 ng of high-quality DNA were cleaved with 0.2 μL *PstI* (10U/uL) at 37 °C for 2 hours. After digestion, the restriction enzyme was deactivated at 85 °C for 20 seconds, and the samples were dehydrated. To perform the ligation reaction, all samples were rehydrated in 6 μL of adapter solutions and incubated at 22 °C for 2 hours in binding mix with T4 DNA ligase (New England BioLabs^®^). For post-ligation reactions, 10 μl from each of the 126 samples (124 progenies + 2 parents) were aliquoted and pooled with 32 samples per group, followed by polymerase chain reaction (PCR) cleanup using the QIAquick PCR Purification Kit^®^ (Quiagen), resulting in four “pre-PCR” GBS libraries. Within each library, PCR amplification was conducted using specific primers for sequencing, using the Illumina platform. PCR purification was performed using the Agencourt AMPure XP PCR purification kit^®^. PCR products were quantified by quantitative PCR, using the KAPA Library Quantification Kit (KAPA Biosystems). For each library, 2 pools of 32 samples were mixed and diluted to 16 pM. For each library, 64 barcoded samples were pooled. The libraries were clustered using TruSeq SR Cluster Kit v3-cBot-HS on the cBOT (Illumina) equipment. The libraries were sequenced using Illumina TruSeq SBS Kit v3-HS on the Illumina HiSeq2500 sequencer (Illumina, San Diego) on two lanes of single-end reads with a read length of 100 bp.

### Read processing and SNP discovery

For all samples, quality trimming was performed with SeqyClean tool v. 1.9.10 (https://bitbucket.org/izhbannikov/seqyclean/) using Phred quality score ≥ 24 and fragment size ≥50. A contaminant database was provided to the program to remove vector, adapter, and other sequence contaminations. The sequence processing was performed using the UNEAK^39^ (Universal Network Enabled Analysis Kit) pipeline with default parameters. UNEAK separates all reads that have an exact match to a barcode plus the subsequent five nucleotides that are expected to remain from a *PstI* cut-site (i.e., 5′… CTGCA’G…3′) but no missing data in the first 64 bp subsequently the barcode. Identical reads were clustered into tags; rare or singleton tags represented by fewer than five reads were excluded to reduce possible sequencing errors. All tags were then aligned pair wisely, and 1-bp mismatches were detected as potential SNPs. To reduce false SNP calls, the UNEAK pipeline applied an error tolerance filter of 0.03.

### Linkage map construction

The UNEAK output files were imported to Tassel v.3.0^50^ in order to apply post-UNEAK filtering. Genotype data were filtered for both parent and progeny samples. Only markers that had no missing genotypes for both parents were retained. Were applied a sample call rate and marker call rate of >80% (i.e., at least 80% SNPs had genotypes called in each sample and a SNP was called in at least 80% of the samples). In addition, only markers that were heterozygous in at least one of the two parents (i.e., AA x AB, AB x AA, or AB x AB) were used for linkage mapping analysis. Next, markers that significantly deviated from Mendelian inheritance, as determined by a chi-squared test (P < 0.001), were excluded. The genetic maps were constructed using the pseudo-testcross strategy. The program R/OneMap was used to assign markers to linkage groups that had a minimum logarithm of odds (LOD) score of 8 and a maximum recombination fraction of 0.35. The program JoinMap4 was then used to construct the map for each linkage group, using the regression mapping algorithm and Kosambi mapping function.

### Analysis of syntenic relationships

The SNPs in the tambaqui linkage maps were used to analyze the syntenic relationships with zebrafish (*Danio rerio*). The sequence reads that harbored the mapped SNPs were extracted and aligned with the zebrafish genome (*Danio rerio*, Zv9) using Bowtie2 v.2.2.5. To maximize the number of alignments, the parameters -D, -R, -N, -L, and -i were optimized manually (-D 100 -R 7 -N 1 -L 20 -i S,1,0.01). Sequences with multiple alignments were removed. The Circos^51^ software was used to plot the relationship between the zebrafish chromosomes and tambaqui linkage groups.

### Functional annotation

The SNPs that successfully aligned with the zebrafish genome (*Danio rerio*, Zv9) were annotated using the Variant Effect Predictor (VEP) tool v.71^52^. The annotations included a range of variant types such as intronic, downstream gene, upstream gene, synonymous, missense, intergenic, splice region, and non-coding transcript.

## Additional Information

**How to cite this article:** Nunes, J. R. S. *et al*. Large-scale SNP discovery and construction of a high-density genetic map of *Colossoma macropomum* through genotyping-by-sequencing. *Sci. Rep.* **7**, 46112; doi: 10.1038/srep46112 (2017).

**Publisher's note:** Springer Nature remains neutral with regard to jurisdictional claims in published maps and institutional affiliations.

## Supplementary Material

Supplementary Information

Supplementary Information

Supplementary Information

## Figures and Tables

**Table 1:** Summary of the GBS reads before and after of trimmed.

	Reads	Total bases (bp)	Trimmed Reads	Trimmed bases	Sequencing depth
Total number	352,425,578	35,242,557,800	285,194,978	27,663,912,870	8.04X
Average per parent	3,870,142	387,014,200	3,264,068	316,614,596	8.60X
Average per offspring	2,779,720	277,972,000	2,247,313	217,989,361	8.03X

**Table 2:** Identification of SNPs from the tambaqui (*Colossoma macropomum*).

SNP	Nucleotide substitution	SNP number	Proportion
Transitions	A-G	28,744	0.42
	C-T	15,288	0.22
	—	44,032	0.64
Transversions	A-C	9,767	0.14
	A-T	7,963	0.12
	C-G	3,483	0.05
	G-T	3,339	0.05
	—	24,552	0.36
Total		68,584	1.00

**Table 3:** Summary of genetic linkage map of tambaqui.

Linkage group	No. of mapped markers	Genetic size (cM)	Average marker interval	Max Gap (cM)
LG1	269	71.0	0.26	4.85
LG2	362	145.7	0.40	8.04
LG3	316	119.2	0.38	4.54
LG4	287	83.1	0.29	3.39
LG5	243	106.0	0.44	10.62
LG6	316	110.2	0.35	7.89
LG7	242	97.8	0.40	4.72
LG8	328	125.1	0.38	8.67
LG9	362	122.2	0.34	5.19
LG10	331	123.2	0.37	5.59
LG11	210	93.3	0.44	4.11
LG12	315	101.7	0.32	14.79
LG13	261	113.7	0.44	3.12
LG14	86	71.5	0.83	3.85
LG15	274	96.6	0.35	3.86
LG16	279	101.3	0.36	2.85
LG17	238	117.4	0.49	6.81
LG18	361	88.8	0.25	3.12
LG19	305	118.5	0.39	6.67
LG20	258	114.4	0.44	2.08
LG21	233	77.6	0.33	3.31
LG22	271	127.5	0.47	4.22
LG23	238	94.6	0.40	3.48
LG24	269	91.6	0.34	3.18
LG25	120	70.9	0.59	4.00
LG26	193	99.1	0.51	3.08
LG27	225	129.2	0.57	6.42
Total	7192	2810.9	0.39	5.27

**Table 4:** Annotation of SNPs for each linkage group of tambaqui against the genes from zebrafish genome (ENSEMBL release 84).

LG	VP	Variants Consequences								
		IN	DW	UP	SY	MI	IT	SP	NC	OT
1	52	13	8	8	6	6	4	2	2	3
2	46	16	3	5	1	3	6	5	6	1
3	46	16	4	5	11	5	3	1	—	2
4	41	15	4	3	2	2	7	—	6	2
5	36	12	5	3	4	1	4	2	3	2
6	56	27	3	6	4	4	7	1	3	1
7	46	14	7	6	2	5	7	4	—	1
8	61	24	9	3	8	5	4	2	2	3
9	79	27	13	6	8	11	6	2	3	2
10	55	16	3	8	4	6	8	3	5	2
11	33	13	5	4	6	1	3	—	1	1
12	49	22	5	2	3	1	9	—	5	1
13	40	20	9	3	1	3	3	—	1	1
14	24	9	3	1	5	2	1	—	2	—
15	69	18	9	7	12	6	11	—	2	4
16	46	12	6	6	8	2	3	—	2	6
17	33	13	5	2	2	3	2	2	3	1
18	58	20	13	9	5	5	3	1	1	1
19	53	15	7	7	9	4	4	2	1	3
20	38	17	2	6	4	2	4	2	—	1
21	47	14	7	5	8	4	4	2	1	2
22	50	24	7	7	4	2	4	1	2	3
23	51	24	4	5	5	3	3	4	3	1
24	49	19	9	6	3	3	7	—	1	—
25	20	7	3	3	1	2	2	—	2	—
26	25	13	3	3	—	—	5	—	—	1
27	34	11	4	5	7	1	4	1	1	1
All	1237	450	159	133	136	90	128	36	58	44

LG: linkage group; VP: variants processed; IN: intron variant; DW: downstream gene variant; UP: upstream gene variant; SY: synonymous variant; MI: missense variant IT: intergenic variant; SP: splice region variant; NC: non-coding transcript variant; OT: others variants.
